# Sorafenib and Mesenchymal Stem Cell Therapy: A Promising Approach for Treatment of HCC

**DOI:** 10.1155/2020/9602728

**Published:** 2020-06-14

**Authors:** Saieh Hajighasemlou, Mohsen Nikbakht, Saeedreza Pakzad, Samad Muhammadnejad, Safoora Gharibzadeh, Milad Mirmoghtadaei, Fariba Zafari, Iman Seyhoun, Jafar Ai, Javad Verdi

**Affiliations:** ^1^Tehran University of Medical Sciences (TUMS), Tissue Engineering & Applied Cell Sciences, Tehran, Iran; ^2^Iran Ministry of Health and Medical Education, Food and Drug Control Laboratory (FDCL), Tehran, Iran; ^3^Tehran University of Medical Sciences, Hematology, Oncology & Stem Cell Transplantation Research Center, Tehran, Iran; ^4^Tehran University of Medical Sciences (TUMS), Cancer Biology Research Center Tehran, Tehran, Iran; ^5^Pasteur Institute of Iran, Department of Epidemiology and Biostatistics, Research Centre for Emerging and Reemerging Infectious Diseases, Tehran, Iran; ^6^Tehran University of Medical Sciences (TUMS), Tehran, Iran; ^7^Qazvin University of Medical Science, Cellular and Molecular Research Center, Qazvin, Iran

## Abstract

Hepatocellular carcinoma (HCC) is the fifth most commonly diagnosed cancer and the second most common cause of cancer-related death worldwide. Sorafenib (Sora) is used as a targeted therapy for HCC treatment. Mesenchymal stem cells (MSCs) are applied as a new approach to fight malignancies. Drug resistance and side effects are the major concerns with Sora administration. The effect of using the combination of sorafenib and MSCs on tumor regression in xenograft HCC models was evaluated in this study. *Methods and Materials*. Human hepatocellular carcinoma cell lines (HepG2) were subcutaneously implanted into the flank of 18 nude mice. The animals were randomly divided into six groups (*n* = 3); each received Sora (oral), MSCs (IV injection), MSCs (local injection), Sora + MSCs (IV injection), Sora + MSCs (local injection), or no treatment (the control group). Six weeks after tumor implantation, the mice were scarified and tumoral tissues were resected in their entirety. Histopathological and immunohistochemical evaluations were used to measure tumor proliferation and angiogenesis. Apoptotic cells were quantified using the TUNEL assay. *Results*. No significant difference was found in the tumor grade among the treatment groups. Differentiation features of the tumoral cells were histopathologically insignificant in all the groups. Tumor necrosis was highest in the hpMSC (local) + Sora group. Tumor cell proliferation was reduced in hpMSC (local) + Sora-treated and hpMSC (IV) + Sora-treated mice compared with the other groups. Apoptotic-positive cells occupied a greater proportion in the Sora, hpMSC (IV) + Sora, and hpMSC (local) + Sora groups. *Conclusion*. A combination of chemotherapy and MSC can yield to more favorable results in the treatment of HCC.

## 1. Introduction

HCC is the fifth most common malignant tumor and contributes to about 800,000 deaths globally per annum [1–3]. Only a small fraction of patients with HCC are candidates for curative treatments, such as surgical resection, liver transplantation, or radiofrequency ablation [4]. Although numerous novel strategies have been proposed to treat HCC [5], including cell-based therapies, the disease remains challenging to combat.

Sorafenib is the only FDA-approved drug that is administered as the first line systemic therapy in advanced HCC [6, 7]. It is a multitargeted molecule that exerts its effect through inhibition of proliferation and angiogenesis of tumor cells via its multi-kinase inhibitory function. However, due to complexity and heterogeneity of the HCC tumor cells, the overall mean survival achieved through sorafenib therapy is less than one year. In addition, considering its adverse effects and being costly, Sora underscores the requirement for other novel therapeutic approaches [8]. Combination therapies with Sora and other therapeutic agents have been therefore suggested to enhance its effectiveness [9–11].

MSCs have become an attractive subject of investigation for treatment of HCCs [12, 13]. They are suitable candidates for cancer therapy due to their multipotency and potential to differentiate into various cell lineages [14], immunoregulatory effects [15], and finally their chemotactic properties that allow them to reside in tumor-contaminated regions [16, 17]. MSCs also induce their effects through upregulation of several proapoptotic genes and downregulation of various antiapoptotic proteins [18]. These cells were shown to have the capacity to both engraft in the liver of carcinoma-bearing BALB/*c* mice and differentiate into hepatocyte-like cells. Furthermore, they can induce tumor cell necrosis [19]. There exists, however, an opposite view regarding the effects of MSCs. They may in fact enhance the growth and metastatic potential of tumoral cells [20, 21]. Further investigations are required to understand the mechanisms underlying such effects.

This study aimed to investigate the antiangiogenic properties of sorafenib and the potential of MSCs alone or in combination with each other to induce tumor apoptosis in a nude mice model of HCC.

## 2. Materials and Methods

### 2.1. Reagents

A total of 21.6 mg of Sora powder (purchased from American LC LAB Company) was dissolved in 150 *μ*L DMSO and 850 *μ*L of sterile physiologic serum to obtain 1 mL of the solution containing 21.6 mg Sora, 50 *μ*L of which was equal to our desired dose of 60 mg/kg.

### 2.2. Cell Culture

HepG2 cell lines were purchased from the National Center for Biological and Genetic Resources of Iran and cultured in RPMI-1640 media supplemented with penicillin (100 U/mL), 10% fetal bovine serum (FBS), streptomycin (100 *μ*g/mL), and then incubated in standard condition (at 37°C, 5% CO_2_ atmosphere, and 95% humidity). Human placenta-derived MSCs were obtained from a single healthy donor [22], cultured in high glucose DMEM media with the conditions mentioned above and used at early passage (3-4).

### 2.3. Xenograft Model

Eighteen male athymic nude mice (nu/nu; C57BL/6) aged 6 to 8 weeks were obtained from the Omid Institute for Advanced Biomodels. The applied treatments in this study were approved by the Ethical Committee of TUMS. The mice were housed and maintained under optimized hygienic conditions in an individually ventilated cage system. The average temperature of each cage was 23°C with relative humidity of 65%. Animal feeding was with autoclaved commercial diet and water ad libitum, and triple ethical principles of working with animals including reduction, refinement, and replacement were implemented. For HCC tumor implantation, 1 × 10^7^ HepG2 cells were suspended in 100 *μ*L of serum-free medium containing 100 *μ*L Matrigel (Corning: 354277) and then inoculated subcutaneously into both right and left flanks of each mouse. Tumor sites were weekly monitored three times and calculated using Vernier calipers. The volume of tumors was calculated based on a standard formula (length × width^2^ × 0.52). When the tumor progressed into an advanced stage, volume of higher than 200 mm^3^, treatment was initiated. The mice were randomly divided into six groups: Sora (60 mg/kg/day) oral, MSC (IV injection), MSC (local injection), Sora (60 mg/kg/day) + MSC (IV injection), Sora (60 mg/kg/day) + MSC (local injection), and control. Injection of human placenta-derived MSC (5 × 10^5^) in the 2nd and 4th groups was via tail veins and in the 3rd and 5th groups was into the tumor margin, whereas the 6th group (control) received a 50 *μ*L combination solution of DMSO and sterile physiologic serum (with the ratio of 150 to 850, respectively), together with an injection of 100 *μ*L of DMEM in tail veins and another 100 *μ*L in tumor margins. An additional injection of MSCs was given one week later. Sorafenib treatment (once a day) via gavage was initiated 15 days after HCC cell injection. The mice were sacrificed on week 4 postimplantation of tumors, and their tumoral tissues and blood were collected. RNA was later added to both blood samples after isolation of serum and tumor tissues (1 mL per 1000/mm^3^). After washing with physiologic serum, tumor samples were transferred to and kept in formalin buffer.

### 2.4. Analysis of Biochemical Factors

Blood samples were collected from the mice and were centrifuged at 800 RCF. To evaluate liver function, serum was extracted and the levels of alanine aminotransferase (ALT) and aspartate aminotransferase (AST) enzymes and urea were determined. Their levels were measured using an automated biochemical analyzer (Mindray).

### 2.5. Histopathological Study

To evaluate the effect of treatment on the histopathological features of tumor sections, the mice were euthanized and the tumoral tissues were dissected on day 28 after treatment and fixed in 10% neutral buffered formalin and finally processed and embedded in paraffin. The embedded paraffin samples were sectioned in 5 *μ*m thickness and stained with hematoxylin and eosin (H&E). The histological sections were blindly evaluated by an expert pathologist under light microscopy (Olympus, Japan) according to the Edmondson–Steiner grading system (1954) [23] for HCC. Furthermore, any histopathological changes such as inflammatory response, necrosis, hyperemia, and hemorrhage were compared in various groups.

### 2.6. Immunohistochemistry (IHC)

Immunohistochemical study was done on 4 *μ*m-thick paraffined sections for evaluating the proliferating cell nuclear antigen and angiogenesis using monoclonal primary mouse anti-human Ki67 (Biocare Medical, USA; 1 : 200) and anti-human CD34 antibodies (Biocare Medical; USA, 1 : 100), respectively. The proliferative index was recorded as mean percentage of positive cells by counting the number of positive stained cells among 100 nuclei in five randomly high magnification selected fields, at 200×, using computer software Image-Pro Plus®V.6 (Media Cybernetics, Inc., Silver Spring, USA).

The angiogenesis index was recorded by counting the CD34-positive vessels in five fields at 200× magnification, and the findings were expressed as the mean number of vessels ± standard error of mean (SEM). The stained sections without the primary antibody for Ki67 and CD34 were used as negative control.

### 2.7. Terminal Deoxynucleotidyl Transferase (TdT) dUTPNick-End Labeling (TUNEL) Assay

TUNEL assay was used to stain the apoptotic cells undergoing DNA fragmentation [24]. After routine deparaffinization, rehydration, and blocking, the slides were stained with TUNEL using the DeadEnd Fluorometric TUNEL system (Promega) based on the manufacturer's protocol:

The mean number of TUNEL-positive cells was recorded for each group under the light microscope.

### 2.8. Statistical Analysis

The findings were expressed as mean and standard deviations (SD). The differences between groups regarding biochemical factors were evaluated by one-way ANOVA. All statistical analyses were performed with STATA Statistical Software Release 15.0 (StataCorp. 2017. Stata Statistical Software: Release 15; StataCorp LLC., College Station, TX). The *p* values < 0.05 were considered statistically significant.

### 2.9. Sample Size Calculation

According to an accepted rule of thumb for sample size in animal studies [25], any sample size which keeps *E* between 10 and 20 should be considered adequate.

*E* = total number of animals − total number of groups.

In our research, we used 18 animals in 6 groups, so *E* = (18−6) = 12; this lies between 10 and 20.

## 3. Results

### 3.1. Analysis of Biochemical Factors

The mean serum levels of AST, ALT, and urea were all in biologically normal ranges. No significant difference was seen between various groups in terms of these biochemical variables.

### 3.2. Histopathological Study

**Figure 1:**
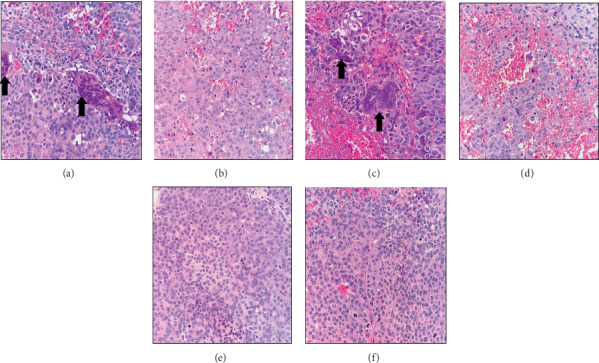
Histopathology feature of HCC xenografts in various treatment groups (H&E stain); thick arrows: pleomorphic tumor giant cells. (a) Ctrl: control, (b) Sora: sorafenib, (c) hpMSC (IV): human placenta-derived mesenchymal stem cells (intravenous administration), (d) hpMSC (local): hpMSC (local administration), (e) hpMSC (IV) + Sora, and (f) hpMSC (local) + Sora. HCC: hepatocellular carcinoma.

The histopathological evaluation of primary tumors showed a solid pattern composed of thick trabeculae and sheath of tumoral cell that were compressed into a compact mass. We did not find any difference in the grading of tumors (using Edmondson–Steiner grading system) among different groups. And the tumoral cells showed histopathologically high grades (III and IV) or poorly differentiated in all treatment groups. In both hpMSC (IV)-treated and control groups, the numerous pleomorphic tumor giant cells were evident histopathologically (Figure 1, thick arrows).

**Figure 2:**
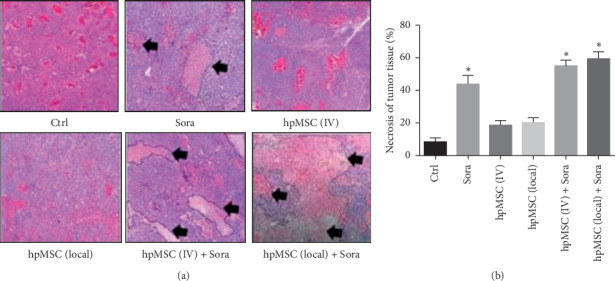
Effects of different treatments on cell necrosis of hepatocellular xenografts over 22 days in nude mice. (a) Histopathologic changes (H&E stain), (b) Calculated percentage of necrotic tissue (%) (+SD). Thick arrows: necrotic areas. Ctrl: control; Sora: sorafenib; hpMSC (IV): human placenta-derived mesenchymal stem cells (intravenous administration); hpMSC (local): hpMSC (local administration); hpMSC (IV) + Sora; and hpMSC (local) + Sora. ^*∗*^*p* < 0.05.

Moreover, different degrees of necrosis were seen in each subject group (Figure 2). The highest severity of necrosis was detected in the hpMSC (local) + Sora-treated mice. These results showed that Sora alone and in combination with MSC significantly induced tumor tissue necrosis compared with the control group. Although, Sora was able to induce tumor cell necrosis, both local and IV administration of hpMSCs could successfully enhance this effect.

**Figure 3:**
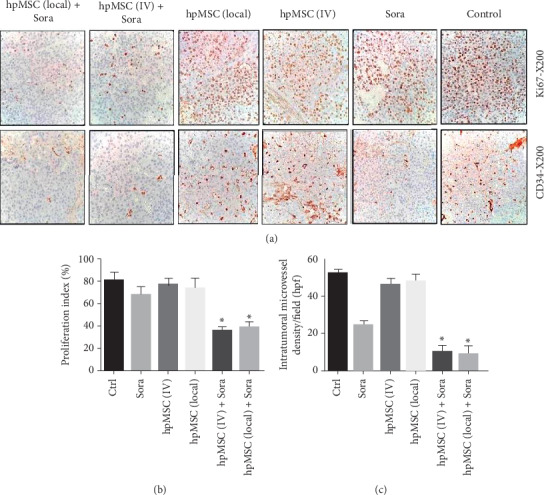
Effects of various treatments on angiogenesis and proliferation of tumoral cells in HCC xenografts in different groups of nude mice using CD34 and Ki67 markers. (a) Histopathological presentation, (b) proliferation index (%) (+SD), (c) intratumoral microvessel density/field (hpf). Ctrl: control; Sora: sorafenib; hpMSC (IV): human placenta-derived mesenchymal stem cells (intravenous administration); HCC: hepatocellular carcinoma. ^*∗*^*p* < 0.05.

The proliferation rate of tumoral cells was determined by analyzing the mean percentage of immunopositive tumoral cells for Ki67 as the marker of cell proliferation in five randomly selected sections. As shown in Figure 3, unlike Sora and MSC alone, co-treatment with Sora and MSC (local or IV) significantly reduced tumoral cell proliferation compared with the control group.

### 3.3. TUNEL Assay

**Figure 4:**
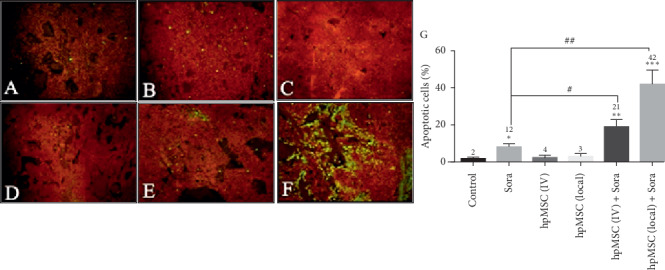
TUNEL staining in the HCC xenograft mice model (magnification, 200×). Green-fluorescent-stained cell nucleus indicates apoptotic cells; red-stained cells represent normal proliferative tumoral cells. (a) Control; (b) Sora; (c) hpMSC (IV); (d) hpMSC (local); (e) hpMSC (IV) + Sora; and (f) hpMSC (local) + Sora, HCC: hepatocellular carcinoma. (g) The diagram shows a quantitative evaluation of apoptotic cells. The mean percentage of apoptotic-positive cells significantly increased in hpMSC (local) + Sora compared with other treatment groups, ^*∗*^*p* < 0.05, ^*∗∗*^*p* < 0.01, and ^*∗∗∗*^*p* < 0.001 vs. control. ^#^*p* < 0.05 and ^##^*p* < 0.01 vs. Sora.

The TUNEL assay was utilized to determine whether the administration of Sora and MSC alone as well as the combination therapy of Sora with MSC can inhibit tumor growth by inducing apoptosis in the tumor cells. The number of apoptotic cells was counted in five high-power fields (400 × magnification), and the mean percentage of apoptotic cells was reported. The Sora alone and in combination with MSC (local or IV) showed significantly higher apoptosis-positive cell count than that of the control group (*p* < 0.01; Figure 4). In addition, the rate of apoptosis in the combination therapy group (MSC + Sora) was significantly higher than that of the Sora alone group.

## 4. Discussion

Available cancer therapies such as chemotherapy, liver transplantation, surgical resection, radiofrequency ablation, immunotherapy, and hormone therapy have different response rates and efficacies due to the vast heterogeneity of HCC [26, 27]. Sorafenib is an approved molecularly targeted therapy that is administered for treatment of patients with advanced HCC through its antiproliferative, antiangiogenic, and proapoptotic functions. These anticancer functions are achieved via targeting some growth factors (GF) such as platelet-derived growth factor receptor (PDGFR), platelet-derived growth factor receptor (PDGFR), and rapidly accelerated fibrosarcoma (Raf) kinases. Systemic treatment with sorafenib can not only improve the overall survival and but delay or inhibit the progression of the tumor; however, the mean survival in this group of patients does not exceed one year and not all patients can tolerate the drug. Therefore, targeting HCC with a combination of Sora plus other therapeutic agents would be a reasonable and promising topic of investigation [9, 11].

In the last decade, cell therapy with MSC has been shown to be a promising approach due to its properties such as easy extraction from various tissues (e.g., adipose tissue, bone marrow, cartilage, umbilical cord blood, and even some solid tumors), fewer ethical concerns, optimal expansion and differentiation into a variety of cell lineages, ability to migrate to injured, inflamed, and cancerous tissues and its immunoregulatory, proregenerative, and antimetastatic effect through production of several GFs and cytokines [13–15, 28].

Apart from these regenerative effects attributed to MSCs, this therapy has the pitfall of promoting revascularization, which may contribute to the progression of malignancies [29]. In addition, MSCs can release various cytokines that influence tumor angiogenesis; these include VEGF and transforming growth factor (TGF) *β*1 [30, 31].

To investigate the likely mechanisms behind the more favorable outcomes observed when Sora was administered in combination with MSCs, we have taken advantage of apoptotic and angiogenesis markers such as Ki67 and CD34, respectively.

The mean serum levels of AST, ALT, and urea as surrogates of liver and kidney function were assessed to ensure the safety of our treatments, none of which showed any significant signs of toxicity.

The degree of necrosis was another variable that was compared between treatment groups to confirm the efficacy of combination therapy. Histopathological evaluation using the Edmondson–Steiner grading system also confirmed that combination of MSCs and Sora increases the degree of necrosis and that the necrotic effect of sorafenib alone was higher than that of MSCs against HCC tumors.

The IHC analysis was used to assess proliferation, angiogenesis, and apoptotic index in tumor tissues. Ki67 is a marker for detecting cell proliferation and has been demonstrated as a prognostic marker for survival in HCC patients [32, 33]. Moreover, the expression of Ki67 is directly proportional to more advanced HCC stages and a poorer differentiation [34]. Here, we have determined the proliferation rate of tumoral cells by counting the mean percentage Ki67-positive cells. The obtained data from IHC showed that the combination of Sora and MSC therapy significantly decreased the proliferation rate of tumoral cells compared with Sora or MSCs alone.

Furthermore, the local injection of MSCs showed higher efficacy in inhibiting the proliferation of tumor cells compared with systemic IV injections.

Analysis of angiogenesis that is a proliferative factor for metastasis and tumor growth can be quantified by microvascular density (MVD). The MVD is evaluated by immunohistochemical assay using an endothelial marker (CD34) that is widely used for assessment of angiogenesis in HCC [35, 36]. Endothelial cells can be derived from human peripheral CD34-positive cells and contribute to angiogenesis in adults [37]. In addition, CD34 is a more sensitive and specific EC marker for detecting of new microvessels in HCC than other commonly used endothelial markers such as CD31 and Von Willebrand's factor (vWF) [38]. We have therefore used CD34 antibodies for this purpose. The higher antiangiogenic effects of sorafenib than that of MSCs are probably due to its inhibitory effect on serine–threonine kinases BRAF and the receptor tyrosine kinase activity of VEGFRs [39]. Furthermore, the results showed that HCC treatment with the combination of MSCs and Sora was more effective in reducing the microvessel density compared with treatment with MSCs or Sora alone, so MSCs, when combined with sorafenib, clearly have antiangiogenic effects on HCC.

TUNEL assay was used to assess treatment response in end cells [40]. The data obtained from the TUNEL assay showed that combination of Sora and MSCs (local or IV) significantly increased the proportion of apoptotic-positive tumoral cells compared with the control group and Sora alone (Figure 4). These results suggested that the combination of Sora and MSCs can significantly reduce the growth of tumor cells by inducing apoptosis.

These concepts are in agreement with other studies assessing combinational therapy with Sora and other therapeutic agents like gemcitabine [41] for HCC treatment that caused a decrease in cell viability and promotion of apoptosis [42, 43]. The antitumor effect of Sora, alone or in combination with other antitumor agents, can be resulted from drug-induced apoptosis [44, 45].

## 5. Conclusion

We conclude that although there is no best way for treatment of HCC, the combination of sorafenib and MSCs has shown a more promising spotlight to achieve more satisfactory results than using sorafenib as a monotherapy. However, more investigations in similar fields would pave the way for an even more extensive clinical trial to take the method to bedside. We propose investigating the variable of drug concentration in the efficacy of such treatment. Future research should also focus on the signaling pathways and the molecular mechanisms involved in both the development of HCC and the effects of MSCs on the progression of tumor cells.

## Data Availability

All data are available upon request to the corresponding author (javad0verdi@gmail.com).

## Abbreviations

HCC: Hepatocellular carcinoma
MSCs: Mesenchymal stromal cells
hpMSC: Human placenta-derived mesenchymal stromal cells
AST: Aspartate aminotransferase
ALT: Alanine aminotransferase
H&E: Hematoxylin and Eosin
Ctrl: Control
Sora: Sorafenib
hpMSC: Human placenta-derived mesenchymal stem cells
IV: Intravenous administration
Local: Local administration
IHC: Immunohistochemistry
TdT: Terminal deoxynucleotidyl transferase
VEGFR: Vascular endothelial growth factor receptor
PDGFR: Platelet-derived growth factor receptor
FDA: Food and Drug Administration
VEGF: Vascular endothelial growth factor
TGF: Transforming growth factor
TopoII*α*: Topoisomerase II alpha
MVD: Microvascular density.
